# Special Issue “Synthesis and Applications of Functionalized Gold Nanosystems”

**DOI:** 10.3390/nano9071046

**Published:** 2019-07-22

**Authors:** Paolo Scrimin

**Affiliations:** Department of Chemical Sciences, University of Padova, via Marzolo, 1-35131 Padova, Italy; paolo.scrimin@unipd.it; Tel.: +39-049-8275276

When I launched this Special Issue, I wrote: “Gold-based nanosystems are among the most interesting systems in the nanoworld because of their broad spectrum of applications, ranging from analyte detection to nanomedicine and the mimicry of enzymes, just to mention a few examples. The size and shape of the nanoaggregates allow one to tune the properties of the gold core, while the introduction of specific functional groups on the passivating monolayer is crucial to modulate the interaction with the surroundings: a target substrate, a protein, and a receptor. In spite of the fact that the literature on the field increases at an exponential rate, I believe that there is space for sound contributions that are able to conjugate the synthesis and applications of these nanosystems on solid experimental bases.” With the publication of 10 original papers and one review, the goal I had in mind has been mostly achieved. Indeed, the contributions that are grouped in this Special Issue provide a good idea of the directions in which the field is moving. The contributions range from the synthesis of special nanosystems [1,2,3] to their utilization for sensing [4,5,6,7] and magnetic resonance imaging [8,9], surface recognition [10], and bioapplications [11].

Thus, Martinez el al. [1] report on the synthesis in an aqueous solution devoid of any surfactant of Au-nanowires of controlled length and reasonably narrow dimensional distribution starting from Au-nanoparticles by taking advantage of the properties of glucosamine phosphate under aerobic conditions and substoichiometric nanoparticle passivation. Their systems are endowed with very peculiar plasmonic properties. Wang, Takara et al. [2] report an effective and straightforward approach to induce directed self-assembly of gold nanotriangles (AuNTs). By taking advantage of the uneven chemical reactivity of AuNT surfaces, they implemented regioselective modifications of the edges and top/bottom surfaces with two different double-stranded DNA (dsDNA) sequences. By means of terminal single-base pairing/unpairing, controlled assembly of dsDNA-mediated AuNTs occurred, eventually evolving in a face-to-face or edge-to-edge disposition. They claim that this approach could be useful for achieving directed self-assembly of other anisotropic nanoparticles. Chen et al. [3] review recent advances on the DNA-based assembly of gold nanostructures and especially emphasize their resulting superior optical properties and principles, including plasmonic extinction, plasmonic chirality, surface-enhanced fluorescence (SEF), and surface-enhanced Raman scattering (SERS). This is a timely contribution highlighting a fast-moving area of research. In the field of sensing, Gaviňa et al. [5] report on gold nanoparticles functionalized with resorcinol moieties that can be used for detecting formaldehyde both in solution and in gas phases. The detection mechanism is based on the color change of the probe upon the aggregation of the nanoparticles induced by the polymerization of the resorcinol moieties in the presence of formaldehyde. A limit of detection of 0.5 ppm in solution was determined. The system may find application whenever the detection of this volatile substance is needed, which is particularly important, considering its toxicity. Mercury is well known for its neurotoxicity. To tackle the problem of its detection, Leopold, Linden et al. [6] present a straightforward reagent-free approach for mercury traces determination using a novel thin film for passive sampling based on gold nanoparticles. Dissolved mercury ions are extracted from a water sample, e.g., river water, by incorporation into the gold matrix in a diffusion-controlled manner. Thus, the amount of mercury accumulated during sampling depends on the mercury concentration of the water sample, the accumulation time, as well as the size of the substrate. Therefore, the experimental conditions can be chosen for any given mercury concentration without loss of sensitivity. An ingenious system has been devised by Li et al. [7] for the detection of hydrogen peroxide. They report the synthesis of Ag–Au bimetallic nanoparticles (Ag–AuNPs) supported on reduced graphene oxide (RGO) with alginate as a reductant and stabilizer. A non-enzymatic sensor was thus constructed through a modified electrode, which showed excellent performance toward H_2_O_2_ with a sensitivity of 112.05 µA·cm^−2^·mM^−1^, a linear range of 0.1–10 mM, and a low detection limit of 0.57 µM (S/N = 3).

Two contributions illustrate the potential of AuNPs in magnetic resonance imaging (MRI). In the first one, Pasquato, Sologan, Padelli et al. [8] describe a proof-of-principle application of gold nanoparticles carrying fluorinated ligands in their monolayer as contrast agents for ^19^F magnetic resonance imaging, displaying high sensitivity because of the high density of fluorine nuclei achievable by grafting suitable ligands on the gold core surface. They studied their approach in a high-field preclinical scanner. By combining Gd(III) chelates, they succeeded in adding a further functional activity to these systems, developing materials also acting as contrast agents for proton magnetic resonance imaging. O’Toole, Keynton et al. [9] exploit the tremendous potential of gold nanoparticles as cancer-targeted contrast agents for diagnostic imaging. The ability to modify the particle surface with both disease-targeting molecules (such as the cancer-specific aptamer AS1411) and contrast agents (such as the gadolinium chelate Gd(III)-DO3A-SH) enabled them to tailor the particles for specific cancer imaging and diagnosis. They were able to distinguish between malignant (MDA-MB-231) and healthy cells (MCF-10A) using a T1-weighted image analysis algorithm based on three-dimensional, deformable model-based segmentation to extract the volume of interest.

Gold nanoparticles may interact with different species in a size-dependent manner, as shown by Grishin et al. [10] For this study, the authors determined the molecular hydrogen adsorption on single gold nanoparticles of various sizes deposited on the surface of highly oriented pyrolytic graphite (HOPG). Hydrogen was dissociatively chemisorbed on the surface of gold nanoparticles with an average size of 5–6 nanometers. An increase in the size of nanoparticles to 10 nm or more led to the inhibition of hydrogen chemisorption and prevented its detection. Finally, Gao et al. [11] show a nice example of bio-application of gold nanoparticles. They studied gold nanoparticles (AuNPs, *d* = 4 ± 1 nm) modified with *N*-isobutyl-l(d)-cysteine (l(d)-NIBC) enantiomers as a model to illustrate the chiral effect on the amylin fibrillation at the nano–bio interface. They showed that both chiral AuNPs could inhibit amylin fibrillation in a dosage-dependent manner, but the inhibitory effect of l-NIBC-AuNPs was stronger than that of d-NIBC-AuNPs. They claim that their results may provide interesting insights for reconsidering the mechanism of peptides amyloidosis at the chiral interfaces provided by biological nanostructures in vivo but also could help design therapeutic inhibitors for anti-amyloidosis targeting and other neurodegenerative diseases as well.

I hope the readers will enjoy browsing through these contributions and will find, perhaps, inspiration for their future research.
